# Efficacy and Safety of Submucosal Intravesical Injection of Platelet-Rich Plasma in the Treatment of Interstitial Cystitis/Painful Bladder Syndrome

**DOI:** 10.1007/s00192-025-06324-6

**Published:** 2025-10-08

**Authors:** M Sherif Mourad, Ahmed Tawfick, Mohamed Kotb, Ismail Mahdy Saleh, Mohamed S. Salim, Younan R. Samir

**Affiliations:** Department of Urology, Faculty of Medicine , Ain Shams University, Cairo, Egypt

**Keywords:** Interstitial cystitis/painful bladder syndrome (IC/PBS), Platelet rich plasma (PRP), Intravesical submucosal injections, O’Leary-Sant Interstitial Cystitis Symptom Index, O'Leary-Sant Interstitial Cystitis Problem Index

## Abstract

**Introduction and Hypothesis:**

The aim of this study was to assess the effectiveness of a single session of submucosal intravesical injections of autologous platelet-rich plasma in the treatment of IC/PBS resistant to conventional methods of treatment.

**Methods:**

This is a prospective one-arm clinical trial that was conducted from April 2021 to April 2023 on 30 patients ranging from 30 to 50 years old with Interstitial Cystitis/Painful Bladder Syndrome symptoms (IC/PBS) not relieved by medical treatment. They were assessed by the O'Leary-Sant Interstitial Cystitis Symptom Index and problem indexes (ICSI) (ICPI). They received a single session of 20 submucosal injections of PRP and were assessed after 1, 3, and 6 months.

**Results:**

Four patients declined to show in the follow-up visits (13.3%) due to personal reasons and were excluded from the final analysis due to incomplete outcome data. Out of 26 patients, 16 patients (61.54%) were considered to have successful results, while treatment failed in 10 patients (38.46%). There was a highly statistically significant decrease in ICPI, ICSI, pain VAS score, frequency, nocturia, and functional bladder capacity (FBC) with a *p* value of < 0.001. Hematuria was observed in three patients (11.5%), while urinary tract infection (UTI) was detected in four patients (15.4%).

**Conclusions:**

A single session of submucosal intravesical injections of platelet-rich plasma (PRP) presents a promising therapeutic option for patients with IC/PBS. The constrains of this research encompass the absence of a placebo arm, further randomized controlled trials are needed to prove its efficacy.

## Introduction

Interstitial cystitis/painful bladder syndrome (IC/PBS) is a chronic, debilitating urological condition with a constellation of lower urinary symptoms accompanied by chronic pelvic pain [1, 2]. The exact pathogenesis of IC/PBS is still doubtful, with multiple theories being proposed, including infection, autoimmune disease, mast cell infiltration, neurogenic mechanisms, defects in the glycosaminoglycan layer, and increased permeability of the bladder to the urinary noxious contents [3, 4]. To date, the primary purpose of IC/PBS treatment is to restore the bladder epithelial function, relieve the pain, prevent recurrence, and improve the quality of life [5, 6]. There are numerous treatments for IC/PBS, including diet therapy, behavioral adjustment therapy, oral medications, intravesical instillations, and intravesical injections [5, 7]. The significant advantage of intravesical treatment is that the substances are applied directly to the tissue to treat and minimize the likelihood of systemic side effects [2, 8, 9].

Platelets modulate the wound healing process, including inflammation and tissue regeneration, by releasing growth factors, cytokines, and extracellular matrix modulators [10]. Platelet-rich plasma (PRP) injections have been widely utilized to treat osteoarthritis through the anti-inflammatory effects of platelet-related growth factors [11]. PRP is rich in several types of growth factors, such as platelet-derived growth factor, epidermal growth factor, and transforming growth factor. Theoretically, with the help of these growth factors, the defective epithelium can undergo proliferation, differentiation, and wound healing, thus reversing the urothelial dysfunction to a normal condition [6, 12]. PRP also secretes several types of cytokines that may initiate a new inflammatory process and may also facilitate the resolution of the unsolved inflammation, thereby eliminating the neurogenic pain caused by the previous inflammation [13].

Utilizing PRP in treating IC/PBS has been recently proposed by several authors. Some authors used PRP as intravesical instillation, others used it as repeated intravesical injections. Some utilized 50 cc of blood to create the PRP while others withdraw 100 cc of blood. We aim to assess the effectiveness of a single session of submucosal intravesical injection of autologous platelet-rich plasma prepared from 50 cc of withdrawn blood in treating IC/PBS resistant to conventional treatment methods [14–18].

### Patients and Methods

We conducted a prospective one-arm clinical trial with a total of 30 patients with inclusion criteria including men and women aged 30–50 years, history of symptoms of bladder pain/discomfort related to bladder filling and accompanied by other symptoms such as frequency and urgency, the symptoms last for not less than 6 months and failed medical treatment in the form of dietary modifications, behavioral adjustment therapy, and at least one of the oral medications such as amitriptyline, antihistamines, and pentosan polysulfate (PPS) for at least 3 months.

Patients with prior intravesical therapy, incomplete medical therapy (receiving therapy for < 3 months), previous bladder or pelvic surgery that could have affected the bladder function, indwelling catheters or ureteric stents, pregnant and lactating female patients, pelvic organ prolapse (POP) assessed by the POP-Q assessment system, untreated urinary tract infection, and elevated serum PSA level were excluded. Abdomino-pelvic ultrasonography was done to exclude patients with suspected bladder stones or masses or backpressure on the kidneys that could suspect ureteric stones.

After obtaining the entire medical history and general examination, including 3-day voiding dairy, IC/PBS symptoms were assessed by the O'Leary-Sant Interstitial Cystitis Symptom Index and Problem Indexes (ICSI) (ICPI). O'Leary-Sant Interstitial Cystitis Symptom and Problem Indexes (ICSI)(ICPI) are widely used scales that assesses the four cardinal symptoms of IC/PBS, i.e., bladder pain, urgency, and frequency during the day and the night. ICSI assess the severity of the symptoms of IC/PBS with a total score ranging from 0 to 20, while the ICPI assesses its impact on daily life with a score ranging from 0 to 16 during the past month.

The pain score was reported by patient self-assessment using a 10-point visual analogue scale (VAS) pain score [19].

The Global Response Assessment (GRA) was used to assess the treatment outcome which is a seven-point symmetric scale used with the following possible responses: markedly worse (−3), moderately worse (−2), slightly worse (−1), no change (0), slightly improved (+1), moderately improved (+2), and markedly improved (+3).

PRP was prepared using the double-spin method, as described by Muthu, using the laboratory centrifuge (Eppendorf™ 5810 R, Hanau, Germany) [20]. Fifty milliliters of whole blood was withdrawn, mixed with an anticoagulant (acid citrate dextrose), centrifuged by a soft spin (100 g for 15 min at 22 °C), followed by a hard spin (1600 g for 20 min at 22 °C). Platelet pellets were added to the normal saline by gently shaking the tube to form 10 mL of sterile PRP. Using this technique resulted in a highly concentrated platelet-rich plasma (PRP) with an estimated platelet count of ~1,000,000 platelets/μL, representing a fourfold increase over baseline levels (assuming an initial whole blood count of 250,000 platelets/μL).

All procedures were performed under general anesthesia, and all patients received a third-generation cephalosporin at the induction of anesthesia.

Patients received 20 submucosal injections of PRP solution; each injection site received 0.5 mL of PRP. The injection needle was inserted about 1 mm into the posterior and lateral walls of the bladder using a 23-gauge needle and a 22-Fr. rigid cystoscope [14].

After the PRP injections, a 16-Fr urethral Foley catheter was left for one night to monitor the urine color, and patients were discharged the next day. Oral antibiotics were prescribed for 3 days.

Patients were followed up in the outpatient clinic after 1 week with urine analysis and culture, then at 1 month, 3 months, and 6 months after the PRP injection. These follow-up visits included detailed medical history, general examination, 3-day voiding dairy to assess the number of urinary frequency, nocturia episodes and functional bladder capacity (FBC) based on the average of a 3-days voiding dairy presented by each patient in each follow-up visit. The results of the Global Response Assessment (GRA), the O'Leary-Sant score (ICSI) (ICPI), and pain VAS were recorded at each follow-up.

The primary end point was assessing the Global Response Assessment (GRA) after 6 months following the PRP injection. We considered patients to have had successful treatment if the reported improvement in the GRA was equal to or greater than two, as reported by Jiang et al. in their study [17]. Otherwise, the treatment was considered to have failed.

### Statistical Analysis

Data were collected, revised, and entered into the IBM SPSS Statistics for Windows, version 23 (IBM Corp., Armonk, NY, USA). The quantitative data were presented as mean, standard deviations, and ranges, while the qualitative variables were presented as numbers and percentages. A complete-case analysis (CCA) was employed, where only participants with complete data across all measured variables were included in the statistical analysis, so of the 30 patients initially enrolled, four (13.3%) were lost to follow-up and excluded from the final analysis due to incomplete outcome data. While CCA may introduce bias if dropouts are related to unmeasured factors, its use was necessitated by lack of patient’s input data. The comparison between more than two paired groups regarding quantitative data and parametric distribution was done by using repeated measures ANOVA test while with nonparametric distribution was done by using Friedman test.

## Results

**Fig. 1:**
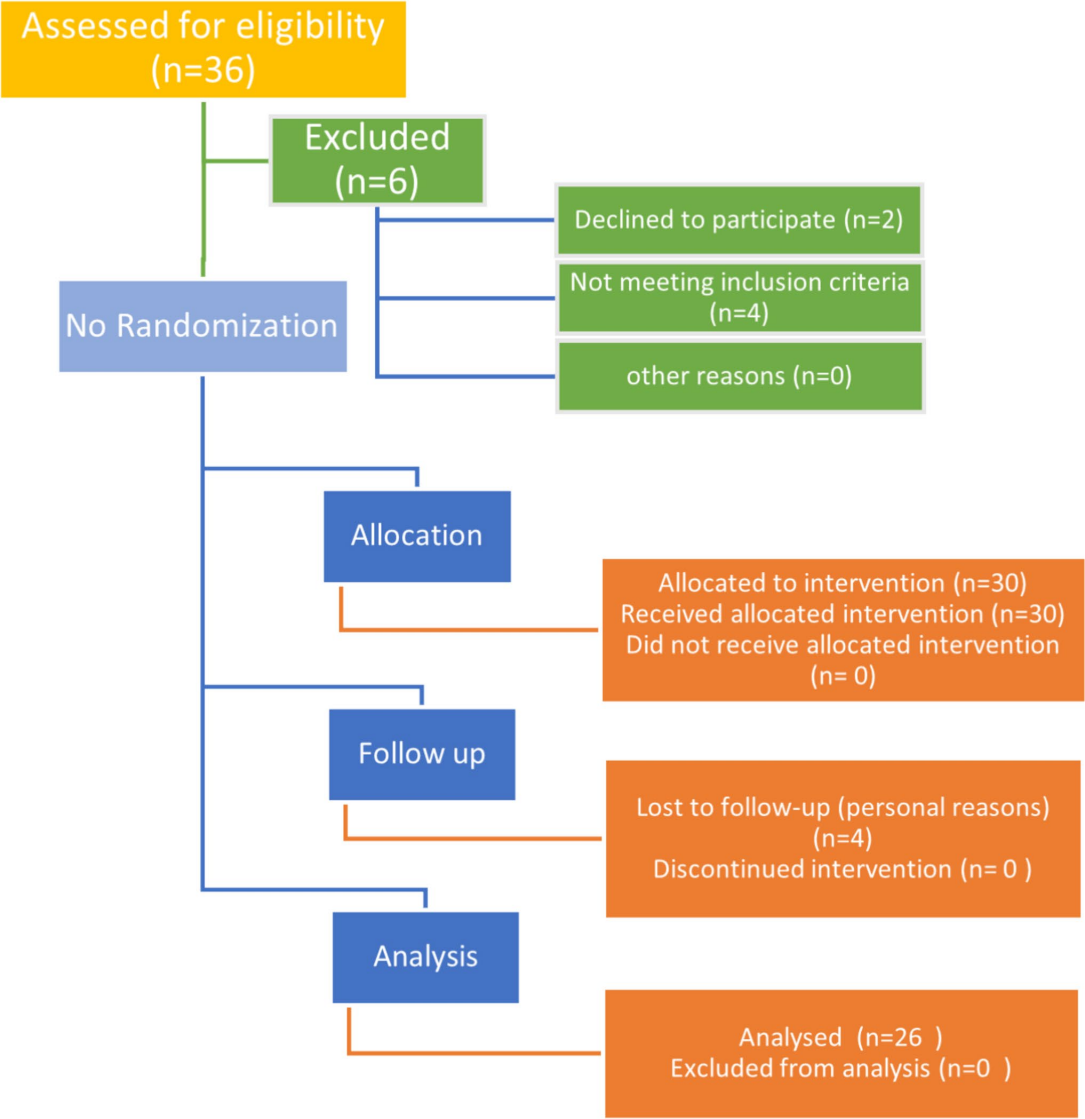
Consort flow chart for our study

As shown in CONSORT diagram (Fig. 1), 30 patients were enrolled in our study after fulfilling our inclusion and exclusion criteria, obtaining informed written consent, four declined to continue and did not show up in the follow-up visits.

**Fig. 2:**
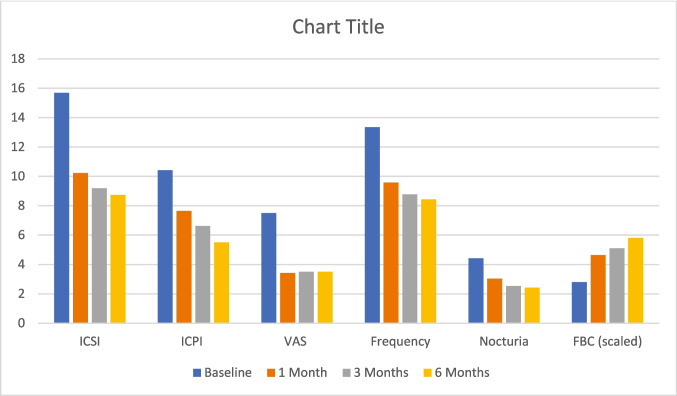
Chart showing the means of O'Leary-Sant Interstitial Cystitis Symptom Index and problem indexes (ICSI, ICPI), 10-point Visual Analogue Scale (VAS) pain score VAS, frequency, nocturia and functional bladder capacity (FBC) (scaled by being divided over 50) at baseline, 1 month, 3 months, and 6 months among the studied patients at 1, 3, and 6 months

Twenty-six patients, 22 women and four men, with an average age of 43.31 ± 4.97 (ranging from 30 to 50) completed our study with the PRP injections. There was a highly statistically significant decrease in ICPI, ICSI, pain VAS score, frequency and nocturia observed after 1, 3, and 6 months compared to baseline, as shown in (Table 1), (Fig. 2) with a p-value of < 0.001.

**Table 1:** O'Leary-Sant Interstitial Cystitis Symptom Index and problem indexes(ICSI, ICPI), 10-point Visual Analogue Scale (VAS) pain score, Frequency, Nocturia and Functional bladder capacity (FBC) at baseline, 1 month, 3 months and 6 months among the studied patients and running Friedman test and Repeated Measures ANOVA test, and GRA response among studied patients at 1, 3 and 6 months

		Baseline	1 month	3 months	6 months	Test value	*P* value	Sig
ICSI	**Median (IQR)**	16 (14−17)	8.5 (7−13)	7 (6−13)	6 (5−12)	49.243 ≠	0.000	HS
	**Mean ± SD**	15.69 ± 1.72	10.23 ± 3.82	9.19 ± 4.31	8.73 ± 4.31			
	**Range**	13–19	6–17	5–17	4–18			
ICPI	**Median (IQR)**	10 (10−11)	7.5 (6−9)	6 (5−8)	4 (3−8)	50.544 ≠	0.000	HS
	**Mean ± SD**	10.42 ± 1.06	7.65 ± 2.45	6.62 ± 2.89	5.50 ± 2.61			
	**Range**	9–13	4–14	4–14	2–14			
VAS	**Median (IQR)**	7 (7−8)	2 (1 − 5)	2 (1−5)	2 (1−5)	46.385 ≠	0.000	HS
	**Mean ± SD**	7.50 ± 0.95	3.42 ± 2.96	3.50 ± 3.04	3.50 ± 3.14			
	**Range**	6–10	1–10	1–10	1–10			
Frequency	**Median (IQR)**	13 (12−14)	9 (8 − 12)	8 (1−12)	7 (6 − 10)	38.733 ≠	0.000	HS
	**Mean ± SD**	13.35 ± 1.32	9.58 ± 2.27	8.77 ± 2.73	8.42 ± 2.76			
	**Range**	11–16	7–14	6–14	6–15			
Nocturia	**Median (IQR)**	5 (4−5)	3 (2 − 3)	2 (1−4)	1 (1 − 4)	41.139 ≠	0.000	HS
	**Mean ± SD**	4.42 ± 1.21	3.04 ± 1.18	2.54 ± 1.23	2.42 ± 1.14			
	**Range**	2–6	1–6	1–6	1–6			
FBC	**Mean ± SD**	139.73 ± 6.3	231.92 ± 41.29	255.31 ± 52.36	290.08 ± 71.3	791.730•	0.000	HS
	**Range**	128–160	125–265	120–288	115–360			
GRA response	−1 (slightly worse)		0 (0.0%)	0 (0.0%)	2 (7.7%)	**Failure**		
	0 (no change)		4 (15.4%)	5 (19.2%)	4 (15.4%)	**Failure**		
	1 (slightly improved)		6 (23.1%)	5 (19.2%)	4 (15.4%)	**Failure**		
	2 (moderately improved)		9 (34.6%)	12 (46.2%)	14 (53.8%)	**Success**		
	3 (markedly improved)		7 (26.9%)	4 (15.4%)	2 (7.7%)	**Success**		

*P* value > 0.05: Non significant; *P* value < 0.05: Significant; *P* value < 0.01: Highly significant; ≠ : Friedman test; •: Repeated Measures ANOVA test, O'Leary-Sant Interstitial Cystitis Symptom Index (ICSI) and O'Leary-Sant Interstitial Cystitis Problem Index (ICPI), 10-point Visual Analogue Scale pain score (VAS), and Global Response Assessment (GRA)

There was also a highly statistically significant rise in FBC comparing baseline with 1 month, 3 months, and 6 months post PRP injection, with a *p* value of < 0.001.

In our study, hematuria was observed in three patients (11.5%), while urinary tract infection (UTI) was detected in the postoperative urine culture 1 week after the procedure in four patients (15.4%).

There was significant improvement in seven patients (26.9%) with GRA 3 after 1 month of injection, which decreased to four patients (15.4%) after 3 months and two patients (7.7%) after 6 months. There was moderate improvement for nine patients (34.6%) at 1 month, increased to 12 (46.2%) patients at 3 months, and reached 14 patients (53.8%) at 6 months. Only two cases (7.7%) had slightly worse symptoms 6 months after the study, while four cases (15.4%) had no change in symptoms, as shown in (Table 1), (Fig. 2).

Two out of four patients who had postoperative UTI and two out of three patients who had postoperative hematuria showed successful study results, indicating that it is not a significant factor in the outcome of our procedure.

**Fig. 3:**
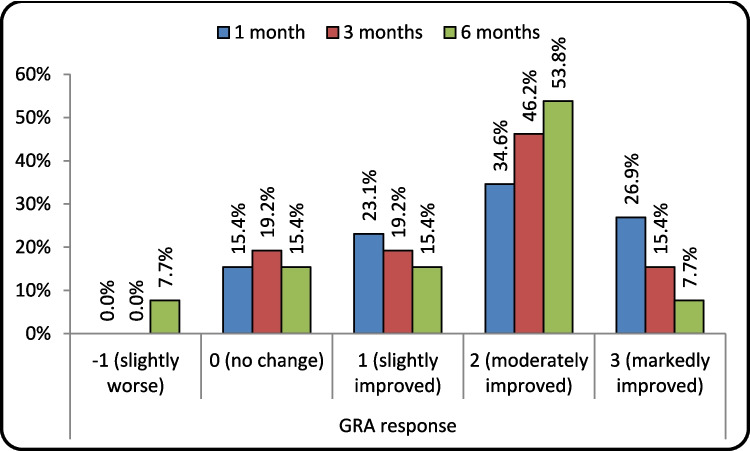
Successful and failed results according to the improvement in the Global Response Assessment (GRA) after 1, 3, and 6 months out of the 26 patients that completed the study

On the basis of the GRA results after 6 months, 16 patients (61.54%) were considered to have successful results out of 26, while treatment failed in 10 patients (38.5%) out of 26, as shown in (Fig. 3).

A nonresponder imputation (NRI) approach was applied to account for missing data, where participants lost to follow-up (*n* = 4) were imputed as treatment failures in the primary end point and the success rate would be 16 patients (53.3%), while treatment failed in 14 patients (46.7%) out of 30.

## Discussion

Our study utilized the submucosal method for PRP injection compared to intravesical instillation, as we thought this ensures better delivery of the PRP into the submucosal layer. Similar to our study, Jhang and colleagues utilized the same method in 2017 and 2018 [14, 17]. Also, Jiang and colleagues in their study in 2020 utilized the same method [15]. Unlike us, El Hefnawy et al. (2022) utilized the intravesical instillation method to inject PRP into the bladder [16].

In our study, We administered PRP through intravesical injection in one session, mirroring Jiang and colleagues in their 2022 study [18]. However, unlike us, Jhang and colleagues in 2017 and 2018 and Jiang and colleagues in 2020 injected PRP monthly for 4 months, whereas El Hefnawy and colleagues in 2022 instilled PRP intravesically weekly for 6 weeks [14–17].

As we tried to evaluate the efficacy of such a new technique, we thought it would be better to have one setting of intravesical injection to assess the efficacy of this technique rather than having repeated injections with repeated anesthesia subjection in each time.

Although four intravesical injections of low-dose PRP have been demonstrated effective, as reported by Jiang in 2022 as the percentage of patients with GRA ≥ 2 after 6 months was higher for those treated with four low-dose PRP injections compared to those treated with a single high-dose PRP injection (67.5% vs 48.3%), yet this necessitates general anesthesia, which places a tremendous burden on the patients [18]. Four monthly injections might also promote complications associated with general anesthesia and bladder injections, such as sore throat, general weakness, hematuria, and UTI. One injection session might decrease the morbidity related to this invasive procedure for IC/BPS. Additionally, removing the requirement for repeated blood sampling that might lead to a drop in hemoglobin, as reported by El Hefnawy et al. [16].

In our study, the number of patients who reported marked improvement of GRA notably declined from seven patients at 1 month to two patients at 6 months post-PRP injection. Further follow-up of the patient may warrant repeating the procedure after 6 months.

Our study found a decrease in ICSI to an average of 10.23 ± 3.82, 9.19 ± 4.31, and 8.73 ± 4.81, a decrease in ICPI values with a mean ± SD of 7.65 ± 2.45, 6.62 ± 2.89, and 5.50 ± 2.61, a reduction in VAS with a mean of 3.42 ± 2.96, 3.50 ± 3.04, and 3.50 ± 3.14, a decrease in frequency with means of 9.58 ± 2.27, 8.77 ± 2.73, and 8.42 ± 2.76, a reduction in nocturia also, with values of 3.04 ± 1.18, 2.54 ± 1.63, and 2.42 ± 1.84 and an increase in FBC with mean ± SD values of 231.92 ± 41.29, 255.31 ± 52.36, and 290.08 ± 71.3 at 1 st, 3rd, and 6th months post-procedure. Similar to us, Jhang in 2017 and 2018, Jiang in 2022, and El Hefnawy in 2022 also reported comparable results [14–18].

Regarding GRA, we found that 61.54% of patients who completed the follow -up visits, had a successful result at 6 months where 14 patients (53.8%) reported moderate improvement and two patients (7.7%) reported marked improvement following our single injection session. On the other hand, ten patients were counted as failures where two (7.7%) patients reported slightly worse outcome, four patients (15.4%) reported no change, and four patients (15.4%) reported slight improvement. Jhang observed an increase of ≥ 2 in the GRA for 54% and 67.5% following the 4th PRP injection in 2017 and 2018, respectively, while, in 2022, El Hefnawy reported a GRA ≥ 2 in 60% [14–18].

Our study found four cases of UTI with a rate of 15.4% in postoperative urine cultures, consistent with El Hefnawy’s 2022 report of three cases with a rate of 15% in positive post-procedure urine cultures. In 2017, Jhang noted that one patient (7%) experienced a UTI. On the other hand, Jhang stated in 2018 that none of the patients examined had a UTI [14–18].

### Limitations

The limitations of this study include the lack of a placebo arm for the placebo effect. In the future, if we need to prove the true therapeutic efficacy of PRP on IC/BPS, a randomized, placebo-controlled trial is still necessary.

Another limitation of this study is its small sample size. The long-term effects, the exact timing required to repeat the procedure, various methods of PRP preparation, the need for monthly booster doses, and the viability of non-autologous PRP remain unknown. Therefore, adequately powered prospective randomized trials comparing the current technique with other submucosal injections or other agents are warranted.

## Conclusion

We concluded that a single session of submucosal intravesical injections of platelet-rich plasma (PRP) prepared from 50 cc of withdrawn blood presents a promising therapeutic avenue for patients with Interstitial Cystitis/Painful Bladder Syndrome (IC/PBS). Significant improvements in patient symptoms were documented, underscoring the potential of PRP in promoting urothelial regeneration and reducing IC/PBS symptoms.

## Abbreviation

IC/PBS: Interstitial cystitis/painful bladder syndrome
PRP: Platelet rich plasma
BOO: Bladder outlet obstruction
CT: Computed tomography
PSA: Prostatic specific antigen
POP: Pelvic organ prolapse
POP-Q: Pelvic Organ Prolapse Quantification System
ICSI: O’Leary-Sant Interstitial Cystitis Symptom Index
ICPI: O'Leary-Sant Interstitial Cystitis Problem Index
VAS: Visual Analogue Scale
GRA: Global Response Assessment
FBC: Functional bladder capacity
UTI: Urinary tract infection
